# Prefrontal spine loss in neuropsychiatric disorders: circuit consequences for cognition

**DOI:** 10.3389/fpsyt.2026.1730869

**Published:** 2026-05-19

**Authors:** Sebastian H. Bitzenhofer, Anne Günther

**Affiliations:** 1 Neural Circuit Physiology, Center for Molecular Neurobiology Hamburg, University Medical Center Hamburg-Eppendorf, Hamburg, Germany; 2 Institute of Developmental Neuroscience, Center for Molecular Neurobiology Hamburg, Hamburg Center of Neuroscience, University Medical Center Hamburg-Eppendorf, Hamburg, Germany

**Keywords:** dendritic spines, neurodevelopmental disorders, neuropsychiatric disorders, prefrontal cortex, pyramidal neurons

## Abstract

Dendritic spine loss on superficial layer pyramidal neurons in the prefrontal cortex (PFC) has emerged as a central neuropathological feature across a range of neuropsychiatric disorders. This is particularly significant as the PFC serves as a critical hub for higher-order cognitive functions such as working memory, attention regulation, and decision-making, which are often disrupted in neuropsychiatric disorders. These cognitive functions rely on the integration of local and global inputs within prefrontal microcircuits, with dendritic spines acting as key structural substrates underlying this integration. Consequently, spine loss in the PFC might compromise functional connectivity within local prefrontal circuits as well as brain-wide networks, ultimately cascading into circuit dysfunction and impaired cognitive performance. By integrating findings across molecular, circuit, and behavioral levels as well as across species, we highlight why, despite disparate etiologies, this shared pathological feature of prefrontal spine loss may act as a mechanistic link between cellular pathology and the complex cognitive symptoms observed in neuropsychiatric disorders.

## 1 Introduction

**Figure 1:**
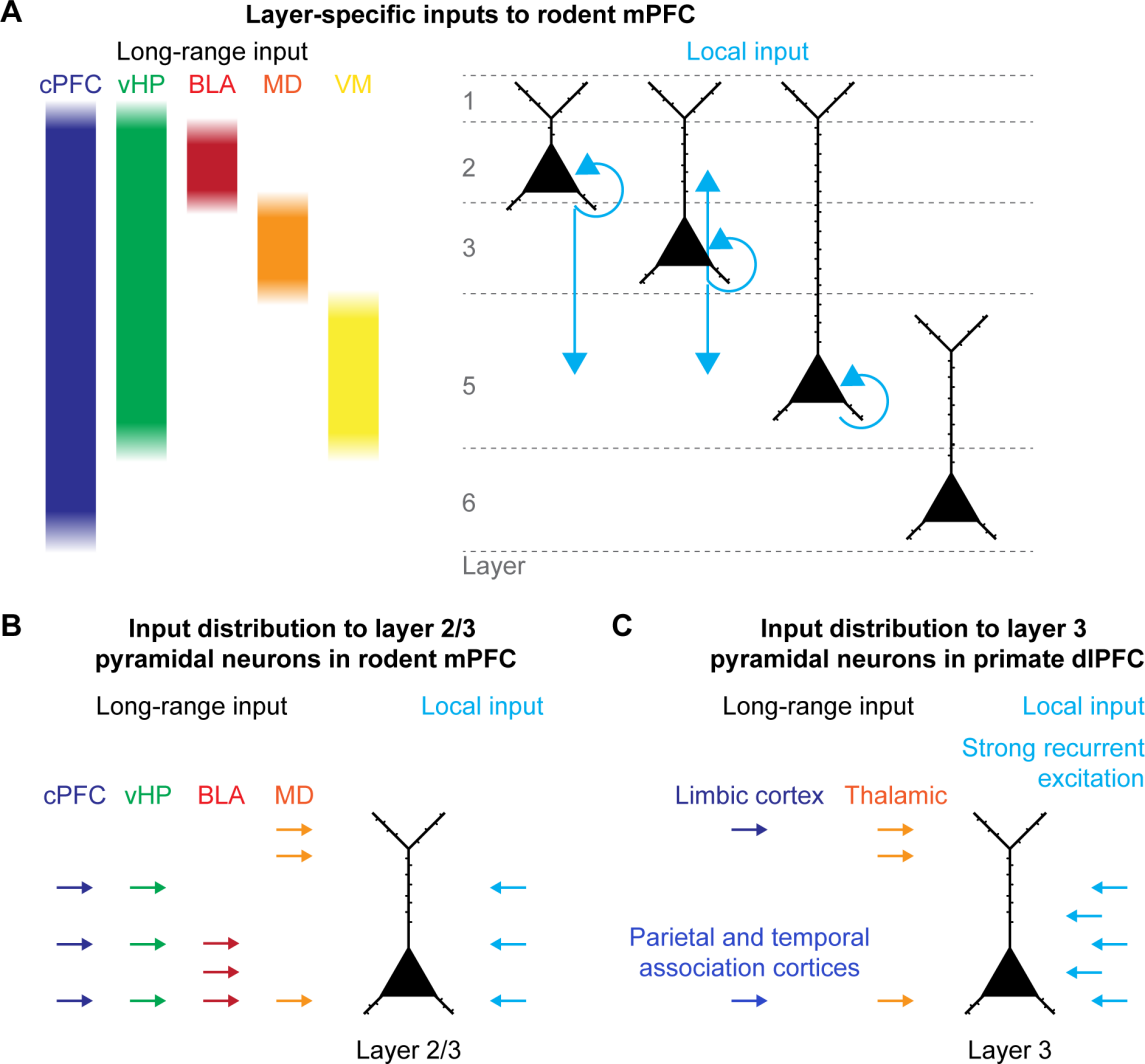
Integration of local and long-range inputs in the PFC. **(A)** Layer-specific long-range (left) and local (right) inputs to pyramidal neurons in the rodent PFC. **(B)** Subcellular distribution of local and long-range inputs to L2/3 pyramidal neurons in the mPFC of rodents. **(C)** Subcellular distribution of local and long-range inputs to L3 pyramidal neurons in the dlPFC of primates. Colors represent input sources highlighted by the text color. (mPFC – medial prefrontal cortex, dlPFC – dorsolateral PFC, cPFC – contralateral PFC, vHP – ventral hippocampus, BLA – basolateral amygdala, MD – mediodorsal nucleus of the thalamus, VM – ventromedial nucleus of the thalamus).

Higher-order cognitive processing is thought to depend on distributed brain networks, with the prefrontal cortex (PFC) playing a central role in coordinating their activity and thus governing a broad range of cognitive functions (1). In particular, the PFC serves as a hub for organizing the flow of information between cortical and subcortical regions and facilitates flexible adjustment of behavior to changing demands. To this end, the PFC integrates inputs from multiple brain regions, including the hippocampus, amygdala, thalamus, and other neocortical areas (2, 3). These inputs preferentially target specific prefrontal layers and are processed within the local recurrent network of the PFC (2, 4, 5) (**Figure 1**). The balance of local and long-range inputs is critical for prefrontal function (6). Particularly, pyramidal neurons in superficial layers of the PFC receive a variety of long-range inputs, which they integrate with local recurrent inputs (2, 7–10). Because of this strategic positioning, superficial-layer pyramidal neurons are especially important for sustaining recurrent activity within the PFC and for the integration of inputs from brain-wide networks.

As spines are the major sites of excitatory inputs to prefrontal pyramidal neurons, altered spine numbers might shift the balance of local and long-range inputs in the PFC and disturb the integration of brain-wide inputs required for the coordination of cognitive abilities. Most cognitive abilities mature throughout development up until the adolescent period, consistent with the prolonged development of the PFC (11, 12). During this prolonged development, spine numbers on prefrontal pyramidal neurons initially increase, followed by a gradual decrease during adolescence until they reach stable levels in early adulthood (12, 13). Recent studies in rodents showed that changes in prefrontal activity during early development (14), thalamic input to the PFC (15), or microglia-mediated synaptic pruning (7) lead to varied forms of long-lasting impairments in the PFC. These effects include altered spine numbers, as well as impaired network activity and cognitive deficits. This prolonged developmental period might underlie the specific vulnerability of the PFC that is associated with neuropsychiatric disorders.

Considering the crucial role of superficial layer prefrontal pyramidal neurons in the integration of inputs for local microcircuit function as well as long-range cortical communication, it is striking that multiple studies on *post mortem* tissue from patients with different neuropsychiatric disorders have identified aberrations in neuronal architecture, specifically in the form of altered spine density or dendritic complexity on superficial layer pyramidal neurons in the dorsolateral PFC (dlPFC) (9, 16, 17). This shared feature among otherwise heterogeneous neuropsychiatric conditions might present a key aspect for improving our understanding of the processes underlying hallmark neuropsychiatric symptoms, such as cognitive impairment.

In this review, we summarize findings of altered spine numbers on prefrontal pyramidal neurons from patient studies, as well as from animal models mimicking a range of neuropsychiatric disorders. We particularly discuss potential aspects from altered spine number to cognitive symptoms commonly described for these disorders. For a broader perspective on the developmental, genetic, and molecular mechanism, we want to point the reader to excellent reviews covering these specific topics (9, 18–20). By combining evidence from structural alterations to circuit dynamics and behavior, we aim to outline how spine pathology in superficial layer pyramidal neurons of the PFC may provide a link between cellular abnormalities and complex cognitive symptoms.

## 2 Dendritic spine loss on prefrontal pyramidal neurons: a common pathological thread

Altered spine densities on pyramidal neurons in superficial layers of the PFC present a convergent phenotype among patients with neuropsychiatric disorders and might thus underlie shared pathological features such as cognitive deficits (9, 11). Here, we summarize key findings on aberrations of prefrontal spine densities from human data and animal models of different neuropsychiatric disorders (**Table 1**).

The comparison of data from different species comes with the challenge to identify homologous areas which is particularly challenging for the PFC. The common definition of the PFC across mammalian species is the part of the cerebral cortex that receives input from the mediodorsal nucleus of the thalamus (21). However, the massive expansion of the PFC in primates and the presence of a granular part of the PFC in primates that is absent in rodents make it difficult to define clear homological subdivisions across species (22). Particularly for the primate dlPFC, no clear homolog area exists in rodents. However, based on structural and functional comparisons, the medial PFC (mPFC) is considered the rodent homolog of the primate dlPFC (22, 23). This limitation should be kept in mind for translational investigations of the PFC. Anyways, spine alterations in superficial layer pyramidal neurons in primate dlPFC and rodent mPFC have been identified across a range of neuropsychiatric disorders.

Due to the above-mentioned limitation of the absence of a clear homolog area for the primate dlPFC, most rodent studies focused on the mPFC or subdivisions of the mPFC. For better readability, we refer to L2/3 and PFC when describing similar findings across species. The details about the specific parts of the PFC and layers for spine alterations are mentioned in **Table 1**.

**Table 1:** Neuronal architecture aberrations on pyramidal neurons in L2 and L3 of the PFC in patients and animal models of neuropsychiatric disease.

Disorder	Species	Model	Area	Layer	Neuroarchitecture	Reference
SCZ	Patient	–	BA10, 11, 45	3	Spine density ↓	(24)
	Patient	–	BA46	3	Basal spine density ↓	(25)
	Patient	–	BA32	3, 5	Basal and apical spine density ↓	(26)
	Patient	–	BA46	3	Basal spine density ↓, dendritic length ↓	(27)
	Patient	–	BA11	3	Basal dendritic length ↓ and branching ↓	(28)
	Patient	*-*	PFC	–	Spine density ↓	(29)
	Mouse	*Setd1a*	mPFC	2/3	Spine density ↓	(30)
	Mouse	*Glun2c*	mPFC	2/3, 5	Basal spine density ↓	(31)
	Mouse	*Disc1*, poly(I:C)	mPFC	2/3	Spine density ↓, dendritic branching ↓	(7)
	Mouse	*Df(16)A*	mPFC	2/3	Spine density ↓	(32)
	Mouse	*Arhgap10*	mPFC	2/3	Spine density ↓	(33)
	Mouse	Poly(I:C)	mPFC	2/3	Spine density ↓, dendritic length ↓ and branching ↓	(34)
	Mouse	*Arhgap10*	mPFC	2/3	Spine density ↓	(35)
	Mouse	*Shank3*	PFC	2/3	Spine density ↓	(36)
MDD	Patient	–	PFC	2, 3	Spine density ↓	(37)
	Rat	Repeat stress	mPFC	2/3	Apical branching ↓	(38)
	Rat	Repeat stress	ACC, PL	2/3	Apical dendritic branching ↓ and length ↓	(39)
	Rat	Mild stress	mPFC	2/3	Apical dendritic branching ↓ + length ↓	(40)
	Rat	Repeat stress	ACC, PL	2/3	Apical spine density ↓, dendritic length ↓	(41)
	Rat	Variable stress	PL	2/3	Apical spine density ↓, dendritic length + branching ↓	(42)
	Rat	Chronic stress	mPFC	2/3	Apical dendritic branching ↓ + length ↓	(43)
RS	Patient	–	BA6	3	Apical dendritic branching ↓	(44)
	Patient	–	BA6	3, 5	Basal dendritic length ↓	(45)
	Patient	–	BA10	3	Apical dendritic branching ↓	(46)
FXS	Mouse	*Fmr1*	PL, IL	3	Apical and basal spine density ↑, immature	(47)
BPD	Patient	–	BA46	3	Basal spine density ↓, dendritic length ↓	(27)
	Patient	–	BA46	3	Spine density ↓, dendritic length ↓	(48)
	Mouse	*Akt*	mPFC	2/3	Basal spine density ↓	(49)
ASD	Patient	–	BA9, 10	all	Dendritic branching ↓	(50)
	Patient	–	BA9	2	Spine density ↑	(51)
	Patient	–	PFC	–	Spine density ↓	(52)
	Marmoset	VPA	BA8b, 9	3	Spine density ↓ (at birth), spine density ↑ (at 6M)	(53)
	Mouse	*Cntnap2*	mPFC	2/3	Basal and apical spine density ↓	(54)
	Mouse	*C58/J*	mPFC	2/3	Apical spine density ↓	(55)
	Mouse	*Rfwd2*	PL	2/3	Spine density ↓	(56)
	Rat	LPS	mPFC	all	Spine density ↓, dendritic length ↓	(57)
	Mouse	VPA	mPFC	2/3	Spine density ↓	(58)
	Mouse	*Shank3*	PFC	2/3	Spine density ↓	(36)
	Mouse	*Dyrka1*	PL	2/3	Dendritic branching ↓	(59)
	Mouse	*Ank2*	mPFC	2/3	Apical spine density ↑	(60)
	Beagle	*Shank3*	PFC	2/3	Spine density ↓, dendritic branching ↓	(61)

SCZ, schizophrenia; ASD, autism-spectrum-disorder; MDD, major depressive disorder; RS, rett syndrome; FXS, fragile x syndrome; BPD, bipolar disorder; *Set1da*, SET domain-containing 1A; *Glun2c*, glutamate ionotropic receptor NMDA type subunit 2C; *Disc1*, disrupted-in-schizophrenia 1; poly(I:C), polyinosinic,polycytidylic acid; *Arhgap10*, Rho GTPase-activating protein 10; *Shank3*, SH3 and multiple ankyrin repeat domains 3; *VPA*, valproic acid; *Cntnap2*, contactin-associated protein 2; *Rfwd2*, ring finger and WD domain 2; LPS, lipopolysaccharide; *Ank2*, ankyrin-2; *Fmr1*, grafile x messenger ribonucleoprotein 1; Akt, protein kinase B; *Dyrka1*, dual specificity tyrosine-phosphorylation-regulated kinase 1a; BA, Broadmann area; ACC, anterior cingulate cortex; PL, prelimbic cortex; IL, infralimbic cortex.

### 2.1 Schizophrenia

Schizophrenia (SCZ) is a complex, heterogeneous disorder that is characterized by a gradual decline in mood and social functioning, psychotic symptoms, and cognitive impairments (62). SCZ was among the first neuropsychiatric disorders identified to present with altered neuroanatomy of pyramidal neurons in specific layers of frontal cortical regions (24). Since then, *post mortem* studies on tissue from SCZ patients have consistently revealed a marked loss of dendritic spine density and dendritic complexity of pyramidal neurons of the dlPFC, particularly in layer (L) 3 (25–28), while other layers remain unaffected (63). Frontal spine loss has been recently confirmed with PET-imaging in early-course schizophrenia patients (29). The selective vulnerability has long been hypothesized to indicate impaired synaptic stability and dysfunctional synaptic pruning, potentially going all the way back to early development (64, 65). Additionally, spine density peaks earlier in L3 (at ~ 2 years of age) than in L5 (at ~ 9 years of age) of the human dlPFC (13). In combination with comparatively less substantial synaptic pruning in L5 and L6 during adolescence than in L3 (66), this might explain the exacerbation of L3 neuron vulnerability during specific phases of development. Furthermore, prefrontal L3 pyramidal neurons in primates show developmental and molecular features which render them particularly vulnerable (67).

Numerous studies in animal models mimicking aspects of SCZ have been conducted, aiming to assess which processes might underlie this specific vulnerability, identifying genetic as well as environmental stressors as potential contributors (7, 11, 30–36, 67, 68). For instance, heterozygous knockout of *Setd1a*, a histone methyltransferase that has been linked to SCZ (69), leads to reduced spine densities on L2/3 pyramidal neurons in the PFC of mice, accompanied by deficits in working memory, social interaction, and prepulse inhibition (30). Furthermore, assessing synapse-targeting risk factors as a potential underlying cause of spine vulnerability, homozygous knockout of the NMDAR subunit *Glun2c* was shown to result in a reduction of basal spines in L2/3 pyramidal neurons in the PFC, as well as deficits in prepulse inhibition, highlighting the potential impact of excitatory-inhibitory imbalance due to NMDA receptor dysregulation in SCZ (31). Similarly, heterozygous truncation of *Disc1*, a scaffolding protein essential for cellular signaling, in combination with an environmental stressor during pregnancy, leads to a reduction of spine densities and dendritic complexity of L2/3 pyramidal neurons of the PFC during early development, accompanied by behavioral deficits (7). A similar reduction of spine densities on L2/3 pyramidal neurons in the PFC and impaired cognitive abilities have been reported for the *Df(16)A* mouse model which mimics the human 22q11.2 deletion syndrome (32). Notably, in the *Disc1* and *Df(16)A* model, the deficits can be reversed by transient application of an anti-inflammatory agent, highlighting a potential role for inflammation in SCZ, as well as the potential for rescue of deficits at suitable time points early in development (7, 32).

### 2.2 Major depressive disorder

Major depressive disorder (MDD) is a mood disorder that is characterized by anhedonia and decreased interest in pleasurable activities, impacting concentration, appetite, and sleep (70). *Post mortem* studies of tissue from MDD patients revealed reduced dendritic spine density in L2 and L3 of the dlPFC (37). Imaging studies in human subjects reported an association between reduced synaptic density and depression severity (71). Stress-induced spine loss in the prefrontal and cingulate cortex contributes to impaired emotional regulation (72). Mechanistically, MDD-induced spine loss is accompanied by downregulation of synaptically enriched proteins as well as upregulation of specific transcription factors modulating the expression of synaptic proteins, suggesting synaptic weakening as a possible driver of dendritic remodeling (37). Stress models in rodents mimicking aspects of MDD replicate these findings, demonstrating spine loss in prefrontal L2/3 pyramidal neurons following repeated stress (38, 39, 41, 42). Notably, a marked pattern of reduced dendritic length and dendritic branching complexity focused on apical dendrites was also observed in numerous studies after mild, repeated, variable, or chronic stress (38–43). These structural alterations are accompanied by functional impairments in working memory and cognitive flexibility (38). The observed pattern of differential effects on apical *vs*. basal dendrites might have a crucial impact on the resulting behavioral and functional deficits.

### 2.3 Bipolar disorder

Bipolar disorder (BPD) is characterized by cyclic elevations in mood that are interspersed with depressive episodes (73). Impairments in attention and executive functions have been reported in the majority of BPD patients and persist even during euthymic phases (74). Similar to SCZ, *post mortem* investigations of the dlPFC of BPD patients revealed reductions in dendritic spine density and dendritic length of pyramidal neurons, specifically in L3 (27, 48). In mice, functional depletion of Akt, a serine/threonine kinase that has been linked to BPD, leads to reduced spine density on basal dendrites of pyramidal neurons in the infralimbic area, accompanied by deficits in recognition and context memory (49). Notably, treatment with the mood stabilizer lithium, which has been shown to promote dendritic growth and spine formation in cultured neurons (75), successfully reverses neuroarchitectural deficits in BPD patients, indicating a degree of plasticity and potential for therapeutic recovery (48).

### 2.4 Autism spectrum disorder

Autism spectrum disorder (ASD) is a neurodevelopmental disorder that is characterized by impaired communication and social interaction, as well as repetitive patterns of behavior (76). While a strong genetic basis has been described for autism, the specific genetic causes identified in different patients are heterogeneous, implicating a large number of genes in ASD etiology (77). *Post mortem* studies on tissue from ASD patients are rather sparse and findings are inconsistent, depending on age, layer, and assessed region of the dlPFC (50, 51). Elevated spine densities on L2 pyramidal neurons of the dlPFC in *post mortem* tissue from ASD patients was shown (51), while a reduction in dendritic branching of pyramidal neurons throughout all layers of the dlPFC was also reported (50). It should be noted that a reduction in dendritic material due to reduced dendritic branching might potentially result in a net loss of spines per neuron, even in cases of increased spine densities. However, a possible explanation for these disparate observations was given by Tang and colleagues, reporting impaired pruning in ASD during adolescence compared to controls (78). The developmental progression of spine densities in L3 of the human dlPFC – *i.e.* a sharp increase in spine numbers from birth until a peak at about 2 years of age, followed by a continuous reduction of spines throughout childhood, adolescence, and adult life (13) – in combination with delayed pruning during adolescence in ASD (78), might provide an explanation for developmental phases of increased spine densities in ASD. This idea is corroborated by results showing comparatively lower spine density at birth but higher spine density at 6 months (~human puberty) in L3 of the dlPFC in a valproic acid-induced ASD model in non-human primates (53). Similarly, mice with knockout of *Ank2*, a structural protein linking ion channels to the cytoskeleton that is considered a high confidence gene for ASD, show increased apical spine densities in L2/3 of prefrontal pyramidal neurons during early development, which normalize towards adulthood (60). Furthermore, a recent PET-imaging study showed decreased spine densities on prefrontal neurons in adult ASD patients (52). Notably, an altered ratio of spine types, shifting from mature mushroom spines towards more immature spine types, persists on L2/3 pyramidal neurons of the PFC (60), potentially impacting integration of inputs and circuit integrity despite normalized spine densities. Taken together, data from human and animal studies point towards a reduction of spine size and spine numbers, altered dendritic morphology, but increased density of spines with immature morphology (79).

Despite this genetic complexity, multiple ASD susceptibility genes converge on cellular pathways involved in the organization of postsynaptic sites (80). Numerous studies have been conducted in animal models assessing the impact of synaptic genes identified as risk factors for ASD (36, 54, 56, 60, 61, 81). For instance, knockout of *Cntnap2*, a member of the neurexin cell adhesion protein family that is essential for synapse formation, results in reduced basal and apical spine densities on pyramidal neurons of prefrontal L2/3 pyramidal neurons (54). Additionally, a reduced number of multi-synapse boutons on these neurons indicates reduced synaptogenesis (54). Similarly, heterozygous knock-in of *Rfwd2*, an E3 ubiquitin-protein ligase involved in dendritic development that has been linked to ASD, leads to reduced spine densities on L2/3 pyramidal neurons of the PFC, as well as deficits in communication, social behaviors, and an increase in repetitive behaviors (56). Notably, these phenotypes are more strongly apparent in male mice compared to female mice (56), mimicking reports of sex-dependent effects of ASD in humans (82). These results, as well as the increased presence of microglia in close proximity to dlPFC pyramidal neurons as has been reported in ASD patients (83), emphasize the potential role of impaired synaptic stability and failure of normal developmental synaptic pruning in the pathology of ASD. Overall, a model of impaired connectivity within brain regions (due to altered neuronal architecture) leading to impaired connectivity between brain regions (due to altered impact of experience-driven connectivity) has been proposed for ASD (84).

### 2.5 Cross-disorder considerations

Across neuropsychiatric disorders, a reduction of dendritic spine density on superficial layer pyramidal neurons in the PFC emerges as a common structural phenotype, suggesting that disrupted synaptic connectivity within the PFC may represent a shared substrate underlying cognitive impairments (9, 18–20). However, the mechanisms leading to spine pathology appear to differ across disorders. In SCZ and BPD, spine loss in superficial layer pyramidal neurons has been linked to genetic risk factors affecting synaptic signaling, NMDA receptor function, and intracellular pathways that regulate synaptic stability, potentially promoting excessive synaptic pruning (11, 27, 49, 64, 85). In MDD, spine loss is more closely associated with chronic stress and transcriptional changes that weaken synaptic maintenance and drive activity-dependent synaptic remodeling (17, 37). ASD involves altered developmental trajectories of synapse formation and pruning, which can result in transiently increased spine densities, a shift toward immature spine types, and ultimately spine loss in adulthood (61, 77, 81, 84). Identifying commonalities across neuropsychiatric disorders is fundamentally challenging due to the complexity of their underlying causes and the diversity of their neuronal and cognitive manifestations. Together, these findings suggest that similar alterations in prefrontal spine density can arise from distinct biological processes which may converge on shared points of vulnerability across disorders. Importantly, the developmental timing of these processes appears to shape the resulting phenotype, emphasizing the need to consider spine loss within the framework of circuit maturation (9, 11, 19, 86, 87).

## 3 Disrupted integration and network dynamics: linking prefrontal spine loss to cognitive symptoms

The PFC is widely recognized as a hub for cognitive control, integrating information from a broad array of cortical and subcortical structures. Many of these long-range afferents target superficial layers, forming excitatory synapses on dendritic spines of L2/3 pyramidal neurons (2). The integrity of spines on these neurons is critical for the PFC to process and integrate these inputs. Thus, reduced spine numbers in superficial layers of the PFC, as consistently observed in many neuropsychiatric disorders, may represent a shared pathological feature that directly contributes to cognitive impairments.

At the cellular level, reduced spine numbers in L2/3 pyramidal neurons of the PFC result in a reduction of excitatory inputs in various animal models of neuropsychiatric disorders (30, 36, 54, 59). Reduced excitatory input seems to be at odds with hyperexcitability of cortical networks associated with ASD, as well as a shift in the ratio between excitation and inhibition towards excitation (88, 89). This apparent paradox can be reconciled by considering evidence for a transient increase in spine densities during development. Later reductions in spine numbers might reflect compensatory mechanisms counteracting increased excitability in early life. Given that L2/3 pyramidal neurons are essential for recurrent local activity and for synchronization with distant brain areas, a reduction of excitatory input might have widespread consequences for prefrontal function.

Within the PFC, pyramidal neurons in L2/3 form recurrently connected networks. These excitatory networks generate reverberatory firing that is critical for sustained representations in working memory (90, 91). More specifically, in the primate dlPFC, particularly recurrent circuits of pyramidal neurons in L3 are essential for the maintenance of working memory related activity (92, 93). Spine loss weakens these recurrent networks, leading to unstable or prematurely decaying activity patterns, a mechanism that might underlie working memory impairments in SCZ (93–96). The specific functionality of thin spines, might explain the high vulnerability of L3 pyramidal neurons in the dlPFC and create a structural link to working memory impairment (97–99). Furthermore, reduced spine numbers might contribute to altered network activity in neuropsychiatric disorders, especially in the gamma frequency range (100, 101). In the PFC, activation of superficial layer pyramidal neurons drives local synchronization in gamma oscillations (8, 102). This local synchronization depends on recurrent excitation between L2/3 pyramidal neurons and inhibitory feedback from fast-spiking interneurons, which is impaired in mouse models of neuropsychiatric disorders (7, 8, 102–104). In primates, extensive recurrent connections of L3 pyramidal neurons in the dlPFC underly the generation of persistent activity and their activation is reflected in gamma activity, providing a link between local circuits, population activity, and working memory (105). The early onset of working memory deficits in the prodromal stage of SCZ correlates with early deficits in L3 pyramidal neurons in dlPFC, indicating that dendritic spine loss on L3 pyramidal neurons might be the primary insult in the progression of the disease and that deficits of fast-spiking interneurons reflect compensatory measures (20). Chronic stress, a considerable risk-factor for neuropsychiatric disorders, has been shown to result in a mPFC specific reduction of spines on L2/3 pyramidal neurons and a selective impairment in an attentional set-shifting task in rodents (38). Inhibition of protein kinase C, mediating stress-induced deficits, rescued dendritic spine loss and cognitive deficits, showing a correlation between L2/3 PFC spine densities and cognitive performance (106). Accordingly, a selective impairment of attentional control and disrupted connectivity in the frontal network have been shown in response to social stress in humans (107). Taken together, these observations highlight how structural alterations at the synaptic level can cascade into macroscopic disturbances of network dynamics and cognition.

In addition to local prefrontal networks, abnormal spine densities and morphology might impair the ability of prefrontal pyramidal neurons to integrate converging signals from distributed brain areas. Several long-range inputs target superficial pyramidal neurons in the PFC, such as prefrontal afferents from the basolateral amygdala (BLA), mediodorsal nucleus of the thalamus (MD), ventral hippocampus (vHP), and the contralateral PFC (2, 4) (**Figure 1**). While inputs from the BLA and MD specifically target pyramidal neurons in L2/3, inputs from the contralateral PFC target all cortical layers (4, 108, 109). In contrast, inputs from the vHP preferentially target pyramidal neurons in L5 in the prelimbic subdivision and all layers in the infralimbic subdivision of the PFC in rodents (4, 110). Interestingly, these areas support different functions of cognitive processing, suggesting that reduced communication with the PFC, caused by a reduction of spine numbers in specific populations of prefrontal pyramidal neurons, might alter the balance of these inputs. Thus, we hypothesize that spine pathology in L2/3 may not simply weaken individual connections, but reshape the balance of inputs from distinct brain regions, resulting in biased processing in prefrontal circuits.

While the mechanisms underlying the integration of diverse long-range inputs in the PFC remain poorly understood, projections from individual areas have been characterized in greater detail. The BLA is considered important for emotional memories, particularly fear learning. It projects strongly to L2 pyramidal neurons of the PFC, where it supports the integration of affective and emotional information to guide behavior (111). Attenuated prefrontal integration of amygdalar input, as expected under conditions of spine loss, might contribute to impaired emotional regulation, and is consistent with impaired emotional processing observed in neuropsychiatric disorders, as well as reduced functional coupling between the amygdala and PFC (112–114). In contrast, MD input to the PFC is critical for working memory maintenance and attentional control (115, 116). Prefrontal inputs from the MD preferentially target L3 pyramidal neurons (117). Inhibition of MD disrupts interactions between the thalamus and PFC, as well as working memory performance, consistent with working memory impairments and decreased thalamo-prefrontal coupling in SCZ (118, 119). Adolescent thalamic inhibition results in perturbations of MD-PFC connectivity, causing long-lasting deficits in prefrontal activity and cognitive functions (15, 120). Prefrontal inputs from the vHP terminate in superficial and deep layers and coordinate oscillatory activity in the PFC underlying spatial working memory (110, 121, 122). Disrupted hippocampal-prefrontal synchrony impairs working memory and has been proposed as a core deficit in SCZ (122–124). The distinct targeting of deep and superficial layers by hippocampal inputs in different subdivisions of the PFC requires further investigation. We hypothesize that a disturbance in the balance between these inputs could underlie altered phase-amplitude coupling between oscillatory activity in theta and gamma frequencies proposed as a biomarker for neuropsychiatric disorders (125–127).

In sum, dendritic spines on L2/3 pyramidal neurons of the PFC are key structural substrates for integrating long-range inputs and supporting recurrent local activity. Their disruption across neuropsychiatric disorders destabilizes local and global network dynamics and impairs prefrontal control over behavior. These circuit-level consequences provide mechanistic explanations for deficits in working memory, cognitive flexibility, emotional regulation, and social cognition. By linking superficial-layer spine pathology to altered network dysfunction, a cross-level framework emerges that connects cellular alterations with cognitive symptoms in disorders such as SCZ and ASD.

## 4 Conclusions

L2/3 pyramidal neurons of the PFC have emerged as critical points of vulnerability in neuropsychiatric disorders. Reduced spine densities and reduced dendritic complexity on these neurons, which are critical for local circuit processing and long-range cortical communication, have been linked to hallmark cognitive deficits, such as impaired working memory, attention, and cognitive flexibility. Notably, even beyond neuropsychiatric disorders, prefrontal spine loss remains a pattern in a wide range of neuropathological disorders. For example, Alzheimer’s disease is characterized by a profound loss of spines in L3 of the PFC, correlating strongly with cognitive decline (128, 129). Also, during normal aging, a decline of cognitive functions appears to be linked to spine alterations in the PFC (130).

The mechanisms driving prefrontal spine loss vary among disorders and include aberrant synaptic pruning, neurotransmitter imbalances, altered synaptogenesis and cytoskeletal dynamics, as well as early-life inflammation (18). Unique molecular regulation in superficial layer pyramidal neurons of the primate PFC might also play a crucial role in their apparent vulnerability (98). Stress-induced spine loss has been implicated to contribute to reduced connectivity in the PFC across disorders, as previously summarized in an excellent review (68). Despite the mechanistic distinctions, a common theme of synaptic disruption emerges across disorders, ultimately impairing excitatory microcircuits in the superficial layers of the PFC and thereby compromising its role in cognition.

Therefore, resolving how distinct, disease-specific mechanisms lead to the convergent structural phenotype of prefrontal spine loss offers a powerful framework for bridging molecular pathology with functional and behavioral outcomes. Future, integrative studies leveraging technologies such as iPSC-derived neurons (131) as well as longitudinal and mechanistic studies that clarify the temporal and causal relationships between cellular structure, circuit dynamics, and behavior will be essential for identifying precise intervention windows and mechanisms. Ultimately, understanding how aberrant spine loss shapes circuit function and thereby cognitive performance may pave the way for therapeutic strategies aimed at preserving or restoring dendritic architecture and may hold promise for improving cognitive outcomes across diverse neuropsychiatric conditions.
